# Influence of in situ 20 ± 2/28 ± 2 kHz dual-frequency ultrasonication on enzymolysis kinetics, thermodynamics and antioxidant activity of housefly (*Musca Domestica*) larvae protein hydrolysate

**DOI:** 10.1016/j.ultsonch.2025.107599

**Published:** 2025-10-03

**Authors:** Han Chen, Fan Yang, Zhuofan He, Liurong Huang, Yiming Zhao, Chunhua Dai, Ronghai He, Haile Ma

**Affiliations:** a Key Laboratory of Food Physics Processing in Jiangsu Provincial Universities, Jiangsu University, 301 Xuefu Road, Zhenjiang, Jiangsu 212013, China; b School of Electrical and Information Engineering, Jiangsu University, 301 Xuefu Road, Zhenjiang, Jiangsu 212013, China; c School of Food and Biological Engineering, Jiangsu University, 301 Xuefu Road, Zhenjiang, Jiangsu 212013, China

**Keywords:** Housefly larvae protein, In situ ultrasonication, Enzymolysis, Kinetics, Thermodynamics, Antioxidant activity

## Abstract

In this study, the influence of in situ 20 ± 2/28 ± 2 kHz dual-frequency ultrasonic irradiation on enzymolysis reaction kinetics, thermodynamics and antioxidant activity of housefly larvae protein (HLP) hydrolysate was investigated to clarify the ultrasonication’s promoting mechanism. The use of in situ dual-frequency ultrasound (DU) treatment significantly increased the reaction rate constant (*k*) of HLP enzymolysis by 82.67 %, decreased the Michaelis constant (*K_m_*) by 13.54 %, and increased the maximum reaction rate (*v_max_*) by 6.12 % compared with conventional HLP hydrolysis. The thermodynamic parameters — activation energy (*Ea*), enthalpy (*ΔH*), and entropy (*ΔS*)—decreased significantly by 24.59 %, 23.95 %, and 18.86 %, respectively. However, no significant change in Gibbs free energy (*ΔG*) was observed, which may be attributed to the enthalpy–entropy compensation effect (EECE). The IC_50_ values of HLP hydrolysate obtained with in situ DU treatment (HLPUH) for DPPH, hydroxyl, and superoxide anion radicals decreased from 3.02, 4.11, and 5.25 mg/mL to 2.64, 3.47, and 4.68 mg/mL, respectively, indicating that the in vitro antioxidant activity of HLP hydrolysate (HLPH) was enhanced by ultrasound treatment. According to the results of in vitro HepG2 cell oxidative stress model experiments, HLPUH exhibited better protective effects on HepG2 cells, inhibited intracellular reactive oxygen species (ROS) expression, and enhanced the activities of intracellular oxidases—catalase (CAT), superoxide dismutase (SOD), and glutathione peroxidase (GSH-Px)—compared with the control. HLPUH also promoted HepG2 cell proliferation and reduced the apoptosis rate.

## Highlights

• Employ in-situ dual-frequency ultrasound to accelerate enzymolysis of Housefly larvae protein.
• The reaction rate constant (*k*) increased by 82.67 %, Michaelis constant (*K*_m_) reduced by 13.54 %.
• *Ea*, Δ*H* and Δ*S* decreased but Δ*G* didn’t vary, ascribing to Enthalpy-Entropy Compensation Effect.
• IC_50_ of HLPUH on DPPH, ^•^OH and O_2_^•−^ decreased from 3.02, 4.11 and 5.25 mg/mL to 2.64, 3.47 and 4.68 mg/mL.
• HLPUH exhibited better in vivo antioxidant protective effects on HepG2 cells.

## 1 Introduction

With the growth of the economy and population, people's demand for food is no longer limited to basic sustenance but has expanded to include health and high quality, leading to an increasing need for new sources of protein-rich foods. Edible insects are considered an extremely promising new protein source due to their sustainable production and low carbon emissions [[1], [2], [3], [4], [5], [6], [7], [8]]. Housefly (*Musca domestica*) larvae represent an ideal source of high-quality protein for future food applications owing to their short lifespan, high nutritional value, rapid reproduction, and low rearing requirements [[9], [10], [11], [12]]. While not yet mainstream in human food products, housefly larvae protein represents a promising and sustainable protein source due to its high nutritional value and low environmental footprint. However, the low solubility and other poor functional properties of housefly larvae protein (HLP) limit its widespread use in food products. In recent years, research has shown that enzymatic hydrolysis of HLP can not only improve its solubility but also generate peptides with antibacterial, antiviral, antioxidant, and immunoregulatory activities [[9], [10], [11], [12], [13], [14]].

Since the antioxidant functions of peptides derived from protein hydrolysates have been demonstrated, the preparation of highly antioxidant bioactive peptides from proteins has become a prominent research focus [[15], [16], [17], [18], [19]]. However, traditional enzymolysis methods for producing functional peptides face several limitations, including uneven reaction systems, low reaction rates, inefficient enzyme utilization, and low biological activity of the resulting hydrolysates [20].

Ultrasound, as an emerging non-thermal physical processing technology, offers advantages such as ease of operation, environmental friendliness, energy efficiency, and safety. It has been widely applied in the extraction, enzymolysis, fermentation, and modification of food proteins [[21], [22], [23], [24], [25], [26]]. The use of ultrasound in enzymolysis can enhance enzymatic efficiency and improve the functional properties of hydrolysates [[27], [28], [29]]. Moreover, studies have shown that different modes of ultrasonic treatment—such as single-, dual-, or multi-frequency irradiation—can promote enzymolysis reactions [[30], [31], [32], [33], [34]].

However, in most of these studies, ultrasound is applied primarily to the substrate, with limited attention given to its simultaneous effects on enzymes or the enzymatic reaction process itself [21,25]. Therefore, in situ ultrasonic irradiation, which acts simultaneously on both proteases and substrate proteins, may lead to more significant enhancements in enzymolysis.

In our previous study [14], we optimized the enzymolysis of HLP using in situ ultrasound at different frequencies and combinations in sweeping-frequency mode (20 ± 2, 28 ± 2, 40 ± 2, 20 ± 2/28 ± 2, 20 ± 2/40 ± 2, and 28 ± 2/40 ± 2 kHz). In the sweeping-frequency mode, the ultrasonic frequency varies periodically within a specified range (e.g., 20 ± 2 kHz), which can enhance the cavitation effect and improve enzymolysis efficiency [14]. The dual-frequency combination of 20 ± 2/28 ± 2 kHz was found to yield the highest increases in both peptide production and 1,1-Diphenyl-2-picrylhydrazyl (DPPH) radical scavenging capacity. Nevertheless, the mechanism by which in situ dual-frequency ultrasound promotes enzymolysis remains incompletely understood.

Kinetic and thermodynamic parameters are crucial indicators in enzymolysis processes, reflecting reaction rates and feasibility. Ultrasound induces strong vibrations, shock waves, cavitation, and shear effects, which can alter protein structure and molecular weight, thereby accelerating reaction rates and improving substrate conversion [20,27,30,31]. Studies have shown that these improvements are associated with reductions in Gibbs free energy, activation energy, activation entropy, and activation enthalpy during ultrasonically assisted enzymolysis [[35], [36], [37], [38]]. Ma et al. [39] further reported that ultrasound significantly influences enzymolysis kinetics by increasing the initial reaction rate through a reduction in the Michaelis constant.

Antioxidant peptides play a crucial role in inhibiting the production of reactive oxygen species (ROS) and alleviating cellular oxidative stress. Their mechanisms are primarily manifested in the following aspects: (1) Antioxidant peptides directly scavenge or indirectly suppress the generation of various ROS, reactive nitrogen species (RNS), and malondialdehyde (MDA); (2) By modulating the activities and expression levels of endogenous antioxidant enzymes—such as catalase (CAT) and superoxide dismutase (SOD)—antioxidant peptides effectively enhance the body's overall antioxidant defense capacity [16,[40], [41], [42]].

At present, the main methods for evaluating the activity of antioxidant peptides include in vitro chemical assays, cell-based approaches, and in vivo animal experiments. Common in vitro chemical methods involve determining the scavenging capacity against superoxide, hydroxyl, and DPPH radicals [43,44]. For antioxidant assessment at the cellular level, human hepatoma HepG2 cells are widely used due to their ease of cultivation and well-documented responsiveness to oxidative stress inducers like H_2_O_2_ and *tert*-butyl hydroperoxide (TBH) [15,45].

This research aimed to reveal the mechanism of in situ dual-frequency ultrasound assisted enzymolysis of HLP from the perspectives of kinetics and thermodynamics through a first-order kinetic model, Mie equation, and transition state theory. And the influence of ultrasound on in vitro antioxidation of HLP hydrolysate were also carried out by using chemical testing and HepG2 cell test. The study will lay the foundation for the application of efficient in situ ultrasound technology in enzymolysis of housefly larvae protein.

This research aims to elucidate the mechanism of in situ dual-frequency ultrasound-assisted enzymolysis of HLP using a first-order kinetic model, the Michaelis-Menten equation, and transition state theory from kinetic and thermodynamic perspectives. Additionally, the effects of ultrasound on the in vitro antioxidant activity of HLP hydrolysate were evaluated through chemical assays and HepG2 cell experiments. This study innovatively employs in situ dual-frequency ultrasound to simultaneously enhance enzymolysis kinetics and thermodynamics of housefly larvae protein, significantly improving peptide yield and antioxidant activity. It systematically links ultrasonic mechanisms to both reaction efficiency and cellular antioxidant effects, providing a novel green strategy for efficient biofunctional peptide production. The findings of this study will establish a foundation for the application of efficient in situ ultrasound technology in the enzymolysis of housefly larvae protein.

## 2 Material and methods

### 2.1 In situ dual-frequency ultrasonication assisted enzymolysis of HLP

**Fig. 1:**
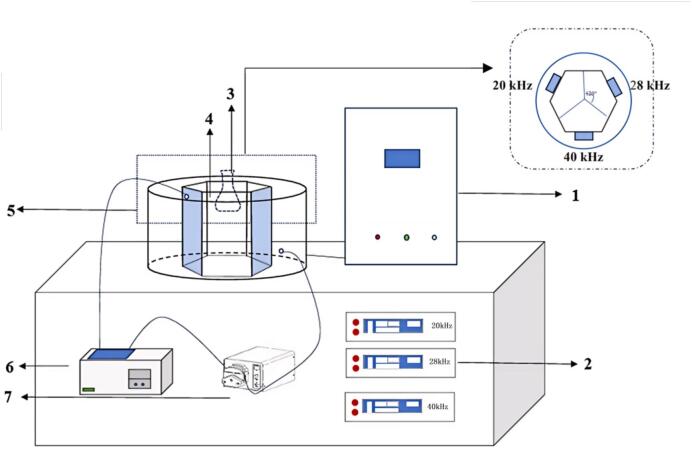
Schematic diagram of ultrasound equipment. (Note: 1-control panel, 2-ultrasonic generator, 3-sample bottle, 4-reaction chamber (4.5 L), 5-ultrasonic converter, 6-constant temperature water bath, 7- peristaltic pump).

The enzymolysis of HLP was assisted using an ultrasonic processor (developed by Jiangsu University, as illustrated in Fig. 1). The interior chamber of the ultrasonic device is hexagonal in design. On every non-adjacent side of the hexagon, sweeping-frequency transducers operating at 20 kHz, 28 kHz, and 40 kHz are installed respectively (refer to the enlarged section in Fig. 1). Based on our previous study [14], the sweeping-frequency amplitude was set to ± 2.0 kHz, with pulse durations of 5 s (on) and 1 s (off). According to the result of our previous study [14], the dual-frequency combination of 20 ± 2/28 ± 2 kHz was selected among various single- and dual-frequency treatments (20 ± 2, 28 ± 2, 40 ± 2, 20 ± 2/28 ± 2, 20 ± 2/40 ± 2, and 28 ± 2/40 ± 2 kHz) due to its optimal enhancing effect on HLP enzymolysis.

Housefly larvae raw material was purchased from Rongfei Ecological Technology Co., Ltd. (Wuhu City, Anhui Province, China). HLP powder was prepared according to the method described by Yang et al. [14]. A 500 mL glass bottle containing 300 mL of HLP suspension (10.88 % (w/v), pH 8.0) was placed at the center of the reaction chamber of the ultrasound equipment (as shown in Fig. 1). The chamber was filled with circulating water maintained at 55 °C for 15 min. Enzymolysis of HLP was conducted following the protocol reported by Yang et al. [14] using an HH-4 digital constant temperature stirring water bath (Jintan Youlian Instrument Research Institute, Changzhou, China) under the following conditions: HLP concentration 10.88 % (w/v), Alcalase 2.4L (Novozymes (China) Biotechnology Co., Ltd., Tianjin, China) dosage 4 % (w/w, enzyme-to-substrate ratio), temperature 55 °C, and duration of 2.57 h.

In this study, the ultrasound intensity was determined by dividing the nominal electrical power input to the transducers by the treatment volume. The dual-frequency ultrasound condition was selected based on the optimal performance identified in our previous study [14]. At the beginning of the enzymolysis process, in situ ultrasonication at a dual frequency of 20 ± 2/28 ± 2 kHz was applied with a total intensity of 42 W/L (21 W/L each for 20 kHz and 28 kHz) to enhance the enzymatic reaction for 25 min. Subsequently, the enzymolysis was allowed to proceed for a total duration of 2.57 h. Samples of the enzymatic hydrolysate were collected at different time points during hydrolysis for measurement of peptide content and antioxidant activity, following the method described by Yang et al. [14].

According to the experimental method described by Su et al. [46], the peptide yield of the hydrolysis products was determined using the Kjeldahl nitrogen determination method in combination with formaldehyde titration. Specifically, an equal volume of 15 % (w/v) trichloroacetic acid (purchased from Sinopharm Chemical Reagent Co., Ltd., Shanghai, China) was added to precipitate and remove high-molecular-weight proteins by centrifugation using a multifunctional desktop centrifuge (Model 5810R, Eppendorf, Germany). The total nitrogen content (*TN*) in the supernatant was then quantified using the Kjeldahl method, while the nitrogen content corresponding to free amino acids (*AN*) was measured by formaldehyde titration [47]. The peptide yield was calculated using the following formula:

(1) $$Peptide\;\;yield\;\left(\%,\;w/w\right)=\frac{\left(TN-AN\right)\times V_{1}}{N_{0}\times W_{1}}\times 100\%$$

In Eq. (1), *TN* represents the total nitrogen content (g/mL) in the hydrolyzed solution after precipitation with trichloroacetic acid; *AN* represents the content of free amino acid nitrogen in the hydrolyzed solution after centrifugation (g/mL); *V_1_* represents the total volume of the hydrolyzed solution (mL); *N_0_* represents the protein content of the enzymatic substrate (determined by Kjeldahl nitrogen determination method) (%, w/w); and *W_1_* represents the mass of the enzymatic substrate (g).

### 2.2 Effect of in situ dual-frequency ultrasonication on kinetics of HFLP’s enzymolysis reaction

(1) Determination of initial reaction rate

Referring to the method described by Zhong et al. [48], the enzymatic reaction kinetics of both ultrasound-assisted and conventional (non-ultrasound) enzymolysis of HLP were investigated. Under both ultrasonic and non-ultrasonic conditions, the enzymolysis reaction conformed to a first-order kinetic model within the first 5 min. Samples were therefore collected at the 5-min mark, and the peptide content in the enzymatic hydrolysate was determined using the Folin phenol reagent method [49]. The initial reaction rate was calculated according to the following equation:

(2) $$v_{0}=\frac{P_{C_{5min}}}{5}$$

In Eq. (2), *v_0_* is the initial reaction rate (mg·mL^−1^·min^−1^) at 5 min of enzymolysis reaction; *P_C_*_5min_ is the peptide content (mg/mL) in the enzymatic hydrolysate at 5-minute.

(2) Determination of enzymolysis kinetics

Referring to the kinetic model established based on the Michaelis–Menten mechanism [50], the enzymolysis reaction kinetics of HLP were analyzed. The kinetic parameters—*K_m_* and *v*_max_—were determined from the slope and intercept of the linear regression curve, respectively. The kinetic model is given as follows:

(3) $$\frac{1}{v}={\frac{K_{m}}{v_{\max}}\frac{1}{S}}_{i}+\frac{1}{v_{\max}}$$

In Eq. (3), *v*_max_ is the maximum reaction rate (mg·mL^−1^·min^−1^), *S_i_* is the initial protein concentration (mg/mL), *v* represents the initial reaction rate of enzymolysis (mg·mL^−1^·min^−1^), and *K_m_* is the Michaelis constant (mg/mL).

(3) Determination of reaction rate constant

The enzymolysis thermodynamics of both HLP hydrolysate (HLPH) and ultrasound-treated HLP hydrolysate (HLPUH) were studied using a first-order kinetic model [33,34]. The reaction rate constant (*k*) was calculated according to the following equation:

(4) $$\ln C=-kt+\ln C_{0}$$

In Eq. (4), *C_0_* is the initial peptide concentration (mg/mL), *C* (mg/mL) is the peptide concentration at a given time *t* (min), and the reaction rate constant *k* (min^−1^) is calculated from the slope of the linear curve of ln *C* versus *t.*

The calculation of the reaction rate constant is according to Eq. (5):

(5) $$\ln\left(V_{\infty }-V_{t}\right)=-kt+\ln V_{\infty }$$

where *V_t_* is the peptide content (mg/mL) in the reaction system at a specific time *t*, *V_∞_* represents the peptide content (mg/mL) when the HLP is completely hydrolyzed in the reaction system, and *k* is the reaction rate constant (min^− 1^). By plotting “ln (*V_∞_* − *V_t_*) ∼ *t*”, the slope of the linear graph represents the reaction rate constant *k*.

### 2.3 Effect of in situ dual-frequency ultrasonication on thermodynamics of HFLP’s enzymolysis reaction

(1) Analysis of thermodynamic parameters of enzymolysis reaction

According to the Arrhenius equation [51], the relationship between the reaction rate constant (k) and the absolute temperature in Kelvin (T) is given as follows:

(6) $$\ln k=-\frac{\mathrm{Ea}}{RT}+\ln A$$

In Eq. (6), *k* represents the reaction rate constant (min^−1^), T is the Kelvin temperature (K), *A* represents the pre exponential factor (min^−1^), *E_a_* is the activation energy (kJ/mol), and R is the molar gas constant (8.314 J/mol·K).

According to Eyring’s transition state theory [52], the thermodynamic parameters of the enzymolysis reaction were calculated using the following equations:

(7) $$\ln\left(\frac{k}{T}\right)=\ln\left(\frac{k_{B}}{h}\right)+\frac{\Delta S}{R}-\frac{\Delta H}{\mathrm{RT}}$$

In Eq. (7), h and k_B_ represent Planck's constant (6.62569 × 10^34^ J/s) and Boltzmann's constant (1.389 × 10^−23^ J/K), respectively. Δ*S* (intercept, J/mol·K) and Δ*H* (slope, kJ/mol) are calculated using the above equation. The calculation formulas for *Ea* (kJ/mol) and Δ*G* (kJ/mol) are as follows:

(8) $$\Delta H=E_{a}-RT$$

(9) ΔG=ΔH-TΔS

### 2.4 Effect of in situ DU on antioxidant activity (in vitro) of HFLP’s hydrolysate

The in vitro antioxidant activities of hydrolysates were evaluated using DPPH, hydroxyl radical (^•^OH), and superoxide anion radical (O_2_^−^) scavenging assays, as these represent key reactive oxygen species relevant to oxidative stress in biological systems.

(1) Determination method of DPPH radical scavenging rate by enzymolysis hydrolysate

Following the method of Zheng et al. [53], 0.5 mL of HLP enzymatic hydrolysate (10 mg/mL) was mixed with 3.5 mL of a DPPH solution (1 × 10^−4^ mol/L in anhydrous ethanol, purchased from Sinopharm Chemical Reagent Co., Ltd., Shanghai, China). The mixture was kept in the dark for 30 min, then centrifuged (4,000 r/min, 8 min), and the absorbance of the supernatant was measured at 517 nm using a UV–Visible Spectrophotometer (Model TU-1810, Beijing Puxi General Instrument Co., Ltd., Beijing, China). The DPPH radical scavenging rate was calculated using the following formula:

(10) $$DPPH\;\;scavenging\;\;rate\;\left(\%\right)=\left[1-\frac{A_{s}-A_{r}}{A_{0}}\right]\times 100\%$$

In Eq. (10), *A_s_*, *A*_r_, and *A_0_* represent the absorbance values of samples containing DPPH, blank samples without DPPH, and DPPH solutions without samples, respectively.

(2) Hydroxyl radical scavenging ability

The experimental procedure was carried out according to the method described by Yu et al. [54]. After mixing the reagents as specified, a suspension of the HLP enzymatic hydrolysate was added. Finally, H_2_O_2_ (purchased from Sinopharm Chemical Reagent Co., Ltd., Shanghai, China) was introduced, and the reaction was conducted at 37 °C for 30 min. The absorbance was measured at 510 nm, and the hydroxyl radical scavenging rate was calculated using the following formula (with water as the blank control):

(11) $$\text{Hydroxyl radical scavenging rate (\textbackslash{}\%)=[1-}\frac{{\text{A}}_{\text{s}}\text{-}{\text{A}}_{\text{r}}}{{\text{A}}_{\text{0}}}\text{][MULSGN]100\%}\;$$

In Eq. (11), *A_s_*, *A*_r_, and *A_0_* represent the absorbance of the enzymolysis product mixed with solvent, enzymolysis product mixed with H_2_O_2_, and solvent mixed with H_2_O_2_, respectively.

(3) Superoxide anion scavenging ability

Referring to the method described by Hu et al. [55], Tris-HCl buffer (trihydroxymethylaminomethane hydrochloride, Sinopharm Chemical Reagent Co., Ltd., Shanghai, China) was first mixed uniformly with disodium ethylenediaminetetraacetate (EDTA-Na_2_, Sinopharm Chemical Reagent Co., Ltd., Shanghai, China). The enzymatic hydrolysate was then added, followed by the addition of o-phenanthroline (Sinopharm Chemical Reagent Co., Ltd., Shanghai, China). After reacting for 10 min, hydrochloric acid (Sinopharm Chemical Reagent Co., Ltd., Shanghai, China) was added to terminate the reaction. The absorbance was measured at 320 nm, and the result was calculated using the following formula:

(12) $$Superoxide\;\;anion\;\;scavenging\;\;rate\;\;\left(\%\right)=\left[1-\frac{A_{s}-A_{r}}{A_{0}}\right]\times 100\%$$

In Eq. (12), *A_s_*, *A*_r_, and *A_0_* represent the absorbance of the enzymolysis product with solvent, the enzymolysis product with solution of pyrogallol, and the mixture of pyrogallol with solvent, respectively.

### 2.5 Influence of in situ DU on the protective effect of oxidative damage in HepG2 cells

(1) Sample preparation

The housefly larvae protein hydrolysate (HLPH) and ultrasound-assisted prepared hydrolysate (HLPUH) were prepared according to the conditions described in Section 2.1. The enzymatic hydrolysate was fractionated using an ultrafiltration system (Pellicon, Millipore Corporation, MA, USA) equipped with membranes having molecular weight (MW) cut-offs of 3 kDa and 10 kDa. During ultrafiltration, the outlet pressure of the membrane module was maintained below 1 bar. This process yielded the following fractions: HLPH-I (MW ≦ 3 kDa), HLPH-II (3 kDa < MW < 10 kDa), HLPH-III (MW ≧ 10 kDa); HLPUH-I (MW ≦ 3 kDa), HLPUH-II (3 kDa < MW < 10 kDa), and HLPUH-III (MW ≧ 10 kDa). All fractions were subsequently concentrated by rotary evaporation and lyophilized. The fraction demonstrating the highest antioxidant activity was selected for further experiments.

(2) Establishment of HepG2 cell oxidative stress model

Referring to the method described by Xiao et al. [56], an oxidative stress model was established. HepG2 cells (obtained from Nanjing Kebai Biotechnology Co., Ltd., Nanjing, China) were treated with Dulbecco’s Modified Eagle Medium (DMEM, Shanghai Datsee Biotechnology Co., Ltd., Shanghai, China) complete culture medium containing different concentrations of H_2_O_2_ (0, 0.2, 0.4, 0.6, 0.8, 1.0, 1.2, 1.4, 1.6, and 1.8 mM). After 6 h of stimulation, the cells were washed with PBS (pursed from Sinopharm Chemical Reagent Co., Ltd., Shanghai, China). The cell survival rate was then determined using a CCK-8 assay kit (New Cell & Molecular Biotech Co., Ltd., Suzhou, China). The H_2_O_2_ concentration resulting in a cell survival rate closest to 50 % was selected as the optimal induction condition. The cell survival rate was calculated using the following formula:

(13) $$Cell\;\;survival\;\;rate\;\left(\%\right)=\frac{A_{1}-A_{0}}{A_{2}-A_{0}}\times 100\%$$

In Eq. (13), *A_1_*, *A_0_*, and *A_2_* represent the experimental wells (culture medium containing H_2_O_2_ and cells CCK-8), the absorbance values of blank wells (culture medium without H_2_O_2_ and cells, CCK-8) and control wells (culture medium without H_2_O_2_ but containing cells, CCK-8).

(3) Cell culture and experimental grouping

Cell Culture:

HepG2 cells (Nanjing Kebai Biotechnology Co., Ltd., Nanjing, China) were cultured in DMEM complete medium containing 100 U/mL penicillin, 100 µg/mL streptomycin, and 10 % (v/v) fetal bovine serum (FBS) at 37 °C with 5 % (v/v) CO_2_ using a CO_2_ incubator ((Vios iDx 165 CO_2_ Incubator, Thermo Fisher Scientific (China) Co., Ltd., Shanghai, China). After adherent growth reached 80–90 % confluence, the cells were passaged four times. Then cells in the logarithmic growth phase were seeded into a 96-well plate at a density of 1 × 10^5^ cells/mL (200 μL per well). The cells were then cultured in an incubator (Vios iDx 165 CO_2_ Incubator, Thermo Fisher Scientific (China) Co., Ltd., Shanghai, China) at 37 °C with 5 % (v/v) CO_2_ for 24 h. When the cell confluence reached approximately 70 %, subsequent experimental procedures were initiated.

Experimental Grouping:

Control group: Cells were treated only with DMEM complete culture medium.

Model group: Cells were first cultured in complete medium for 24 h, followed by stimulation with H_2_O_2_-containing culture medium for 6 h.

Sample group: Cells were incubated with culture medium containing enzymolysis hydrolysates HLPH-I and HLPUH-I for 24 h, respectively, and then stimulated with H_2_O_2_-containing culture medium for 6 h.

(4) Determination of antioxidant enzyme activity in HepG2 cells

Referring to the method of Soltani et al. [57], HepG2 cells were treated following the procedures outlined in Sections 2.5(2) and 2.5(3). The activities of intracellular catalase (CAT), glutathione peroxidase (GSH-Px), and superoxide dismutase (SOD) were measured using corresponding assay kits (the kits were purchased from Nanjing Jiancheng Bioengineering Institute, Nanjing, China).

(5) Intracellular ROS scavenging assay in HepG2 cells

HepG2 cells were treated according to the procedures described in Sections 2.5.2 and 2.5.3. After treatment, the culture medium was discarded. Using ROS assay kit (purchased from Shanghai Beyotime Biotechnology Co., Ltd., Shanghai, China), and following with the instruction of the kit, an equal volume of 2′,7′-Dichlorodihydrofluorescein diacetate (DCFH-DA, purchased from Sinopharm Chemical Reagent Co., Ltd., Shanghai, China) probe was added, and the cells were incubated in the dark for 30 min. After washing with ice-cold PBS, the fluorescence intensity of the cells was measured using an ELISA reader (Infinite M200 Pro, Tecan, Switzerland) at an excitation wavelength of 485 nm and an emission wavelength of 525 nm. Cellular morphology was observed using a fluorescence inverted microscope (Leica DMI 4000B, Wetzlar, Germany). Quantification of fluorescence levels was done with Image J software (National Institutes of Health, USA).

(6) Cell cycle analysis of HepG2 cells

HepG2 cells were grouped and treated according to the methods described in Sections 2.5.2 and 2.5.3. Following the instructions of the assay kit (Shanghai Beyotime Biotechnology Co., Ltd., Shanghai, China), the cell cycle distribution of HepG2 cells was analyzed using a flow cytometer (CytoFLEX, Beckman Coulter, Inc., USA). Data processing was performed with FlowJo software (FlowJo LLC, USA).

### 2.6 Statistical analysis

All experiments were repeated three times independently. Data were analyzed by one-way ANOVA using SPSS 20 software (IBM Corporation, USA), with a significance level set at α = 0.05. Graphs were generated using Origin 2023 software (OriginLab Corporation, USA).

## 3 Results and discussions

### 3.1 Effect of in situ DU on kinetics of HLP’s enzymolysis reaction

(1) The effect of in situ *DU* on the initial reaction rate of enzymolysis

**Fig. 2:**
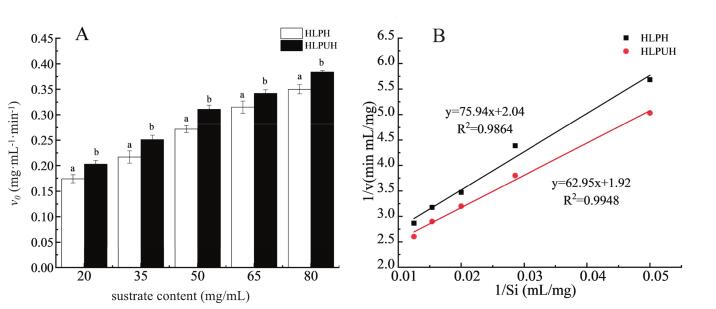
The kinetic parameters of HLPH and HLPUH during enzymolysis. A: Initial reaction rates with different substrate contents; B: The 1/v ∼ 1/Si curves. (Note: different letters in same substrate content in A or same column of the table in B indicate significant differences, *P* < 0.05).

As shown in Fig. 2A, the initial reaction rates of both ultrasound-assisted and conventional (non-ultrasound) enzymolysis increased with rising substrate concentration. Across all concentrations tested, ultrasound treatment significantly enhanced the initial reaction rate of housefly larvae protein hydrolysis compared to the non-ultrasound method, with increases of 16.15 %, 15.71 %, 14.17 %, 8.39 %, and 9.55 % (*P* < 0.05) at different substrate concentration levels.

At low substrate concentrations, the likelihood of collision between enzyme and protein molecules is limited. As the concentration increases, the frequency of enzyme-substrate interactions rises, thereby accelerating the formation of hydrolyzed products [58]. These findings are consistent with those of Wen et al. [29], who reported that dual-frequency ultrasound pretreatment more effectively promoted the enzymolysis of watermelon seed protein compared to single-frequency mode, with the 20/28 kHz combination showing the greatest enhancement.

To evaluate the effect of ultrasound-assisted enzymolysis of HLP on kinetic parameters, the Lineweaver–Burk equation was applied, as shown in Fig. 2B. The 1/v ∼ 1/Si plots for both conventional and ultrasound-assisted enzymolysis exhibited strong linear regression, with coefficients of determination (R2) of 0.986 and 0.995, respectively. The cavitation and mechanical effects of ultrasound induce conformational changes and exposure of active sites in HLP during enzymolysis, thereby accelerating the hydrolysis reaction. These results align with the findings of Wang et al. [59], who reported that ultrasound enhanced the enzymolysis of rapeseed protein by altering its molecular structure, increasing the surface area available for enzyme immobilization, and improving product yield.

The Michaelis constant (*K_m_*) is a key parameter in enzymolysis kinetics, independent of substrate concentration. A lower *K_m_* value reflects a higher enzymatic affinity for the substrate and a faster reaction rate [50]. As calculated from Fig. 2B, ultrasonic treatment increased the *v_max_* of the reaction by 6.12 % (*P* < 0.05) and decreased *K_m_* by 13.54 % (*P* < 0.05). These results indicate that ultrasound enhanced the affinity and binding frequency between housefly larvae protein (HLP) and alkaline protease, thereby accelerating the enzymolysis rate and improving peptide yield. The reduction in *K_m_* (13.54 %) and increase in *v_max_* (6.12 %) indicate that ultrasound enhances both substrate affinity and catalytic turnover. This suggests dual mechanisms: (1) structural unfolding of HLP exposing cleavage sites, and (2) potential enzyme activation via conformational flexibility induced by acoustic forces. This finding is consistent with the report by Dabbour et al. [38] on the ultrasound-assisted enzymolysis of sunflower meal protein, in which a decrease in *K_m_* and an increase in *v_max_* were observed, indicating more favorable conditions for the enzymatic reaction.

The significant enhancement in enzymatic reaction kinetics through in situ dual-frequency ultrasound—evidenced by increased initial reaction rates, reduced Michaelis constant (*K_m_*), and elevated reaction rate constants (*k*)—provides a strong foundation for future research and industrial applications. Further studies should also explore the integration of real-time monitoring and feedback control systems in ultrasonic reactors to dynamically adjust parameters during enzymolysis, ensuring consistent product quality and energy efficiency.

And it is important to acknowledge that the classical Michaelis-Menten model assumes homogeneous aqueous-phase kinetics under ideal mixing conditions, while in situ ultrasound introduces significant physical and mechanical effects—such as cavitation, microstreaming, and continuous alteration of enzyme and substrate conformations—that may challenge these underlying assumptions. Future studies should employ more complex kinetic or reactor models that account for sonomechanical and sonochemical effects to fully decouple biochemical improvements from physical enhancements.

(2) The effect of in situ DU assisted enzymolysis on reaction rate constant

**Fig. 3:**
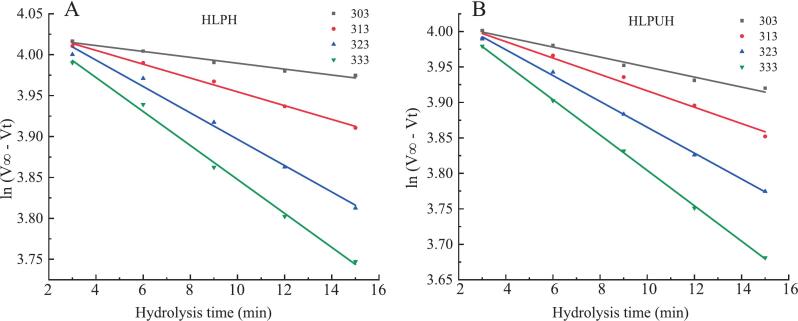
The ln(*V_∞_*-*V_t_*) versus enzymatic time (min) during the enzymolysis of HLPH. (A) and HLPUH (B) at different temperatures (303, 313, 323, 333 K).

The reaction rate constant (*k*) is a key kinetic parameter that directly reflects the velocity of an enzymolysis reaction. Using the Arrhenius equation and transition state theory (TST), thermodynamic parameters were calculated [37,38]. The constant *k*, which is temperature-dependent, plays a central role in these models. Accordingly, we examined the effects of both conventional non-ultrasonic enzymolysis and ultrasound-assisted enzymolysis on the values of *k* across a temperature range of 303 ∼ 333 K. The relationships between ln (V∞ − Vt) and reaction time for enzymolysis with and without in situ dual-frequency ultrasound (DU) assistance at a fixed temperature are presented in Fig. 3.

At all tested temperatures, the curves exhibited strong linear relationships with enzymolysis time, with correlation coefficients exceeding 0.9600. Therefore, a first-order kinetic model was employed to determine the reaction rate constant (*k*), which was calculated from the slope of the linear regression [36]. The values of *k* at different temperatures are summarized in Table 1. As the temperature increased from 303 K to 333 K, both the conventional and ultrasound-assisted enzymolysis groups (Fig. 2, Fig. 2) showed an upward trend in *k* values. The highest reaction rates were observed at 333 K, indicating that this temperature is more favorable for the enzymolysis reaction. At elevated temperatures, the frequency of collisions between enzymes and housefly larvae proteins (HLPs) increases, enzyme activity is enhanced, and the resulting rise in *k* values positively contributes to the efficiency of the hydrolysis process [37,38]. These findings collectively demonstrate that ultrasonic pretreatment significantly enhances enzymolysis efficiency by improving enzyme-substrate affinity, with dual-frequency ultrasound proving particularly effective in optimizing reaction kinetics across varying conditions.

**Table 1:** The reaction rate constants of HLPH and HLPUH.

	*T* (K)	*k* (min^−1^)	R^2^
HLPH	303	0.0036 ± 0.0007^a^	0.9796
	313	0.0073 ± 0.0006^b^	0.9947
	323	0.0141 ± 0.0009^c^	0.9906
	333	0.0208 ± 0.0013^d^	0.9963
HLPUH	303	0.0067 ± 0.0005^b^	0.9796
	313	0.0112 ± 0.0008^e^	0.9947
	323	0.0189 ± 0.0009^d^	0.9907
	333	0.0255 ± 0.0015^f^	0.9951

Note: Different letters indicate significant differences, *P* < 0.05.

Mean values ± standard deviations (n = 3).

Moreover, across the temperature range of 303–333 K, the reaction rate constants (k) for in situ dual-frequency ultrasound-assisted enzymolysis were significantly higher (*P* < 0.05) than those of the non-ultrasound groups, with increases of 82.67 %, 48.70 %, 33.39 %, and 23.04 % (*P* < 0.05), respectively. This enhancement can be attributed to the cavitation and mechanical effects of ultrasound, which disrupt the structure of HLP, promote protein unfolding, and increase the exposure of enzyme binding sites and active regions, thereby facilitating the enzymolysis reaction [20]. These findings are consistent with those of Dabbour et al. [38], who reported that ultrasound treatment significantly increased the *k* value during the enzymolysis of sunflower meal protein and enhanced enzymatic activity compared to conventional methods. The relative improvement in *k* value by ultrasound decreased with increasing temperature (from +82.67 % at 303 K to +23.04 % at 333 K), indicating that thermal energy progressively dominates reaction kinetics, while ultrasound provides maximal enhancement under suboptimal thermal conditions.

### 3.2 Effect of in situ DU on thermodynamics of HLP’s enzymolysis reaction

**Fig. 4:**
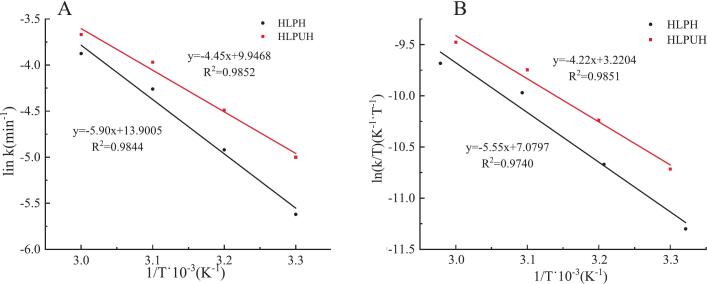
The relationship between ln *k* against 1/T (A) and ln (*k*/T) against 1/T (B) in HLPH and HLPUH during enzymolysis at different temperature.

In enzymolysis reactions, activation energy (*E_a_*) represents the minimum energy required to transition substrate molecules from the ground state to the activated state. *E_a_* enables stable molecules to undergo reaction and thus significantly influences the reaction rate. Typically, *E_a_* values range between 40 and 400 kJ/mol; reactions with *E_a_* below 40 kJ/mol proceed more rapidly [32,33]. Using the Arrhenius equation, *E_a_* was determined from the slope of the linear relationship between ln k and 1/T (shown in Fig. 4) according to Equation 6.

As shown in Table 2, the *E_a_* value for in situ dual-frequency ultrasound-assisted enzymolysis was 36.99 kJ/mol, representing a 24.59 % (*P* < 0.05) decrease compared to that of the non-ultrasound process. This reduction in *E_a_* indicates that the enzymolysis reaction proceeds more readily under ultrasound treatment, which may lead to lower energy consumption and higher product yield. These results align with the findings of Quaisie et al. [60] in their study on sea cucumber protein, where ultrasound was shown to reduce the *E_a_* of the reaction, decrease energy requirements, and enhance enzymolysis efficiency.

**Table 2:** Thermodynamic parameters for HLPH and HLPUH.

	*E_a_*	Δ*H*	Δ*S*	Δ*G*			
	(kJ/mol)	(kJ/mol)	(J/mol·K)	303 K	313 K	323 K	333 K
HLPH	49.05 ± 3.41^a^	46.14 ± 3.87^a^	−171.17 ± 9.84^a^	97.70 ± 1.05^a^	99.40 ± 1.21^a^	101.10 ± 1.05^a^	102.81 ± 1.0^a^
HLPUH	36.99 ± 2.97^b^	35.09 ± 2.56^b^	−202.26 ± 7.61^b^	96.37 ± 0.67^a^	98.39 ± 0.85^a^	100.42 ± 0.64^a^	102.44 ± 1.07^a^
Reduction(%)	24.59	23.95	18.86	n.s.	n.s.	n.s.	n.s.

Note: Different letters in same column indicate significant differences, *P* < 0.05.

n.s. indicates not significant, *P* > 0.05.

Mean values ± standard deviations (n = 3).

Compared to ultrasonic pretreatment of protein substrates prior to enzymolysis, in situ ultrasound irradiation acts simultaneously on both the protein and the protease. This dual exposure can modify the conformational structure of the enzyme and potentially enhance its catalytic activity. However, to prevent enzyme inactivation caused by excessive ultrasonic intensity, the power used for in situ treatment must be considerably lower than that applied in pretreatment methods. For instance, the optimal power for in situ dual-frequency ultrasound in this study was 42 W/L, while Wen et al. [29] and Ding et al. [31] reported optimal pretreatment powers of 100 W/L and 120 W/L, respectively, to achieve the greatest enhancement of enzymolysis. This comparison highlights a key advantage of in situ ultrasonication: its ability to promote enzymolysis efficiently with significantly lower energy input.

According to the Eyring transition state theory, a fitting curve between ln (*k*/T) and 1/T was obtained, and *ΔH*, *ΔS*, and *ΔG* were calculated. As shown in Table 2, compared with non-sonicated enzymolysis, *ΔH* and *ΔS* were significantly reduced by 23.95 % and 18.86 % (*P* < 0.05) after ultrasonication. The results showed that ultrasound altered the conformation of the HLP, leading to the disruption of internal hydrophobic interactions, stabilizing the relationship between proteins and enzymes, causing oxidative changes in amino acid residues and converting enzyme-substrate interactions from lower energy levels to excited states, thereby enhancing enzyme activity [31]. Collectively, these studies confirm that ultrasound-assisted enzymolysis enhances efficiency by reducing activation energy and improving enzyme-substrate affinity.

Compared with non-sonicated enzymolysis, *ΔS* decreased by 18.86 %, indicating that the shear force generated by ultrasound breaks down HLP into smaller fragments, thereby increasing the surface area of the reaction interface, improving the affinity between the enzyme and substrate, and accelerating the enzymatic reaction [36]. Zhao et al. [34] found similar results in the study of mulberry leaf protein, i.e. ultrasound treatment reduced the *ΔS* of the reaction, thereby accelerated the enzymatic reaction.

From Table 2, it can be seen that during the enzymolysis process, *ΔG* increases with increasing temperature (303∼333 K). However, *ΔG* values did not show significant variation (*P* > 0.05) in ultrasound and non-ultrasound assisted enzymolysis. Quaisie et al. [60] reported that very slight reduction (4.66 %∼6.46 %) of *ΔG* in ultrasound treated enzymolysis compared to non-ultrasound. However, no significance analysis of the differences was performed in their study. The observed phenomenon—significant changes in Ea, *ΔH*, *ΔS*, yet no notable variation in *ΔG* during enzymolysis—may be attributed to the Enthalpy–Entropy Compensation Effect (EECE) [61]. The observed invariance in *ΔG* despite significant reductions in *E_a_*, *ΔH*, and *ΔS* strongly suggests the presence of Enthalpy–Entropy Compensation (EECE), wherein the stabilization afforded by the decrease in enthalpy is counterbalanced by a loss in conformational entropy, resulting in minimal net change in Gibbs free energy and reflecting a thermodynamic trade-off underlying ultrasound-induced enzymatic acceleration. Specifically, ultrasonic irradiation accelerates enzymatic reactions by reducing the activation energy, but its concurrent effects on enthalpy and entropy often counterbalance each other, resulting in minimal change in Gibbs free energy. The observed EECE in ultrasound-assisted enzymolysis system—where significant reductions in *ΔH* and *ΔS* yield minimal change in *ΔG*—closely parallels the thermodynamic behavior reported by Jiang et al. [62] for calcium binding to dipeptides (Gly-Tyr/Tyr-Gly). In both systems, EECE emerges as a fundamental mechanism governing molecular interactions under external stimuli (ultrasound or pH/temperature changes). While ultrasound increases collision frequency, it also orients molecules for optimal binding (e.g., via shear-induced alignment). This dual effect explains both the reduced *E_a_* (due to more productive collisions) and reduced *ΔS* (due to loss of rotational/translational freedom).

The thermodynamic improvements achieved through in situ dual-frequency ultrasound—particularly the reduction in activation energy (*E_a_*), enthalpy (*ΔH*), and entropy (*ΔS*)—suggest promising pathways for future research and industrial applications. Further investigations should also explore the broader implications of the Enthalpy–Entropy Compensation Effect (EECE), using advanced spectroscopic or computational methods to characterize ultrasound-induced structural changes in proteins and enzymes at the molecular level.

### 3.3 Effect of in situ DU on in vitro antioxidant activity of HLP’s hydrolysate

In vitro antioxidant activities of HLP’s hydrolysates (with and without in situ DU treatment), i.e. DPPH, hydroxyl radical (^•^OH) and superoxide anion radical (^•^O_2_^−^) scavenging abilities were measured, and their IC_50_ values were also calculated.

**Fig. 5:**
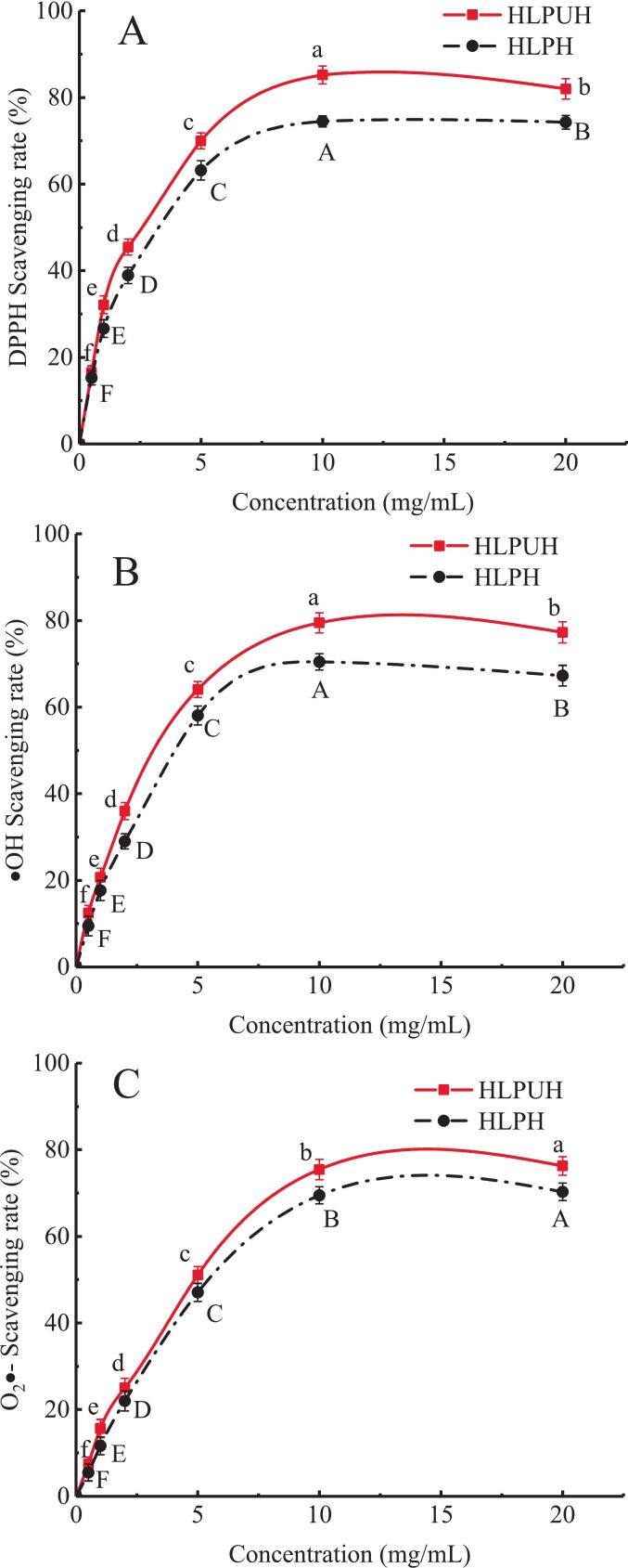
Antioxidant capacity of HLPH and HLPUH. (A: DPPH radical scavenging rate; B: hydroxyl radical scavenging rate; C: superoxide anion radical scavenging rate, different letters indicate significant differences, *P* < 0.05).

As shown in Fig. 5A, the DPPH radical scavenging rates of both HLPH and HLPUH gradually increased with rising concentrations of the hydrolysate and eventually plateaued, reaching maximum values of 75.07 % and 85.15 % (*P* < 0.05), respectively, at 10 mg/mL. The IC_50_ value of HLPUH was 2.64 mg/mL, which is 14.39 % (*P* < 0.05) lower than that of HLPH (3.02 mg/mL), indicating a significant enhancement in the antioxidant capacity of the ultrasound-treated hydrolysate.

These results are consistent with the findings of Tian et al. [63] in their study on wheat germ protein, where ultrasonic treatment significantly improved the antioxidant activity of the hydrolysate. The cavitation effect induced by ultrasound may promote the exposure of hydrophobic groups in HLP, leading to the generation of peptides with hydrophobic amino acid residues at their termini, which are known to contribute to antioxidant potency. Furthermore, Yang et al. [14] reported that dual-frequency ultrasound (20 ± 2/28 ± 2 kHz) was more effective than single-frequency modes in enhancing both peptide yield and DPPH radical scavenging activity. This improvement may be attributed to the increased cavitation nuclei and enhanced mechanical effects under dual-frequency irradiation, which generate a broader frequency range and more intense cavitation, thereby facilitating more efficient enzymolysis and release of bioactive peptides.

The scavenging rates of ^•^OH and ^•^O_2_^−^ of HLPH and HLPUH were shown in Fig. 5, Fig. 5. As the hydrolysate’s concentration increased, ^•^OH and ^•^O_2_^−^ scavenging capabilities of HLPH and HLPUH also increased accordingly, reaching their maximum values at 10 mg/mL, respectively, and finally remained stable. After ultrasound treatment, the IC_50_ values for scavenging ^•^OH and ^•^O_2_^−^ (3.47 and 4.68 mg/mL) of HLPUH decreased by 18.44 % and 12.18 % (*P* < 0.05), compared with those of HLPH (IC_50_ 4.11 and 5.25 mg/mL), respectively. In enzymolysis reactions, the cavitation effect induced by ultrasound reduces the particle size of the hydrolysate, exposes additional hydrophobic groups, and increases the specific surface area of the product. These changes provide more reaction sites for free radical scavenging, thereby enhancing the antioxidant activity of the hydrolysate [64]. Consistent with these findings, Zheng et al. [65] reported that ultrasound treatment significantly improved the antioxidant activity of bovine bone protein hydrolysate, with a maximum increase of 32.57 %.

The significant enhancement in antioxidant activity of HLP hydrolysates via in situ dual-frequency ultrasound treatment opens several avenues for future research and application. Identifying, isolating, and characterizing the specific antioxidant peptides would help clarify structure–activity relationships and support the rational design of highly active antioxidant ingredients.

### 3.4 Influence of in situ DU on the protective effect of oxidative damage in HepG2 cells

(1) Ultrafiltration separations of HLPH and HLPUH

**Fig. 6:**
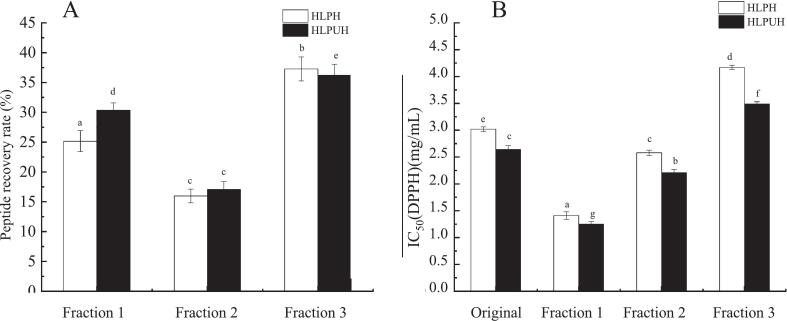
The peptide recoveries and DPPH IC_50_ values of each fraction of HLPH and HLPUH by ultrafiltration. (Fraction 1: Molecular weight ≦ 3 kDa; Fraction 2: 3 kDa < Molecular weight < 10 kDa; Fraction 3: Molecular weight ≧ 10 kDa. Different letters indicate significant differences, *P* < 0.05).

HLPH and HLPUH were fractionated by ultrafiltration into distinct molecular weight ranges: HLPH-I (MW ≦ 3 kDa), HLPH-II (3 kDa < MW < 10 kDa), HLPH-III (MW ≧ 10 kDa); and HLPUH-I (MW ≦ 3 kDa), HLPUH-II (3 kDa < MW < 10 kDa), and HLPUH-III (MW ≧ 10 kDa). The peptide recovery rates and IC_50_ values on DPPH of each fraction after ultrafiltration separation were shown in Fig. 6. During the ultrafiltration process, the residual liquid in the pipeline and ultrafiltration membrane resulted in decreases in peptide recovery rates to 83.64 % and 78.41 %, respectively (Fig. 6A). From Fig. 6B, it can be seen that after ultrafiltration separation, the smaller the molecular weight of the components, the higher their antioxidant activity. Compared with non-sonicated enzymolysis, the antioxidant effect of each fraction was better after ultrasonic treatment. HLPH-I and HLPUH-I components with molecular weights less than 3 kDa had the highest antioxidant activity, with IC_50_ values of 1.41 mg/mL and 1.25 mg/mL on DPPH scavenging, and peptide recovery rates of 25.16 % and 30.37 %, respectively. These findings align with those of Jia et al. [66], who reported a similar inverse relationship between peptide molecular weight and antioxidant activity in silkworm pupa protein hydrolysate. These results collectively demonstrate that ultrafiltration effectively isolates bioactive peptides by molecular weight, with lower-MW fractions consistently exhibiting superior bioactivity (ACE inhibition or antioxidant capacity). Furthermore, ultrasound pretreatment not only enhances the bioactivity of the resulting peptides but also improves peptide recovery rates, highlighting its dual benefit in optimizing both yield and function.

(2) Protective effects of HLPH-I and HLPUH-I on intracellular antioxidant enzymes’ activities in oxidative damaged cells induced by H_2_O_2_

**Fig. 7:**
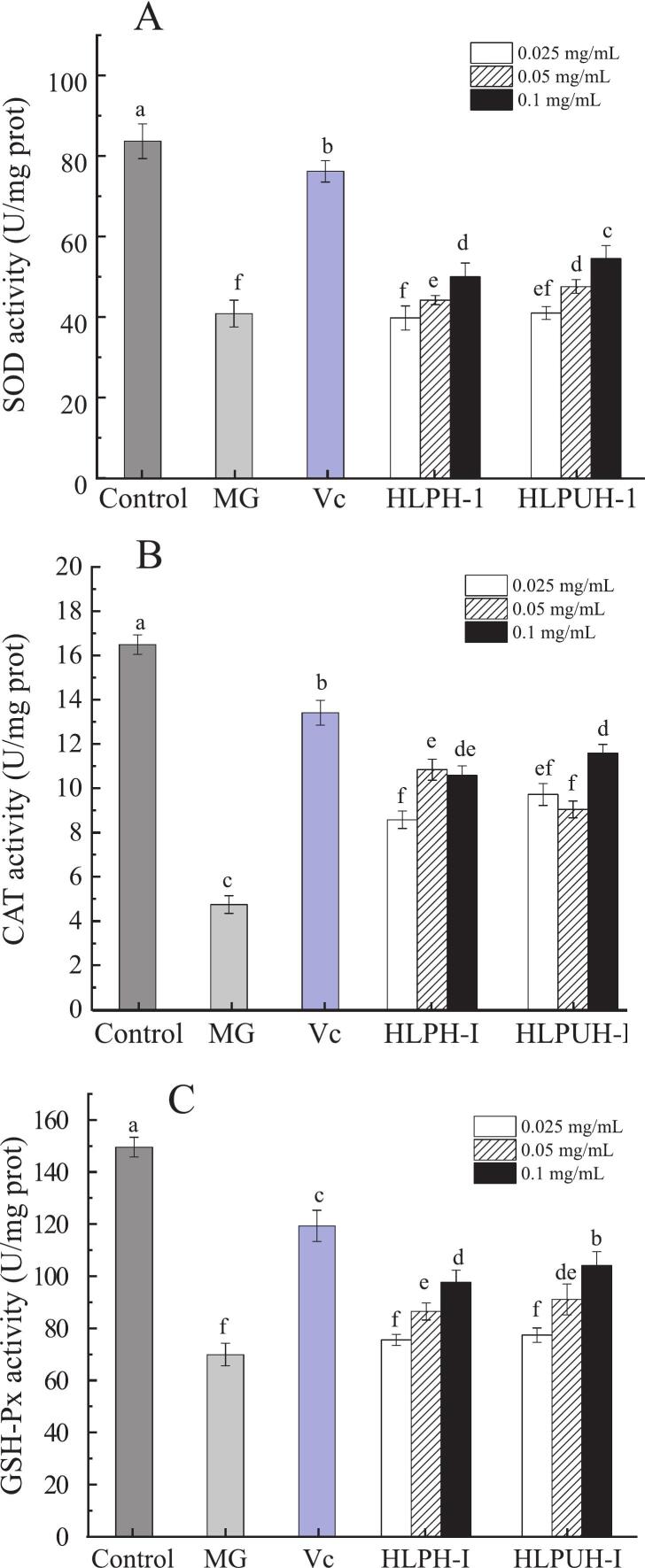
Protective effects of HLPH-I, HLPUH-I and Vc on intracellular antioxidant enzymes. (A: SOD; B: CAT; C: GSH-Px) activities in oxidative damaged cells induced by H_2_O_2_ (Note: MG means model group; HLPH-I, HLPUH-I are HLPH’s and HLPUH’s fractions whose molecular weights lower than 3 kDa, with low concentration (0.025 mg/mL), medium concentration (0.05 mg/mL), and high concentration (0.1 mg/mL), respectively. Using Vc as positive control with concentration of 8 μmol/L. Different letters indicate significant differences, *P* < 0.05).

In normal cells, superoxide dismutase (SOD) degrades highly reactive superoxide radicals by converting them into oxygen and hydrogen peroxide, thereby contributing to free radical clearance. As illustrated in Fig. 7A, HLPH-I, HLPUH-I are HLPH’s and HLPUH’s fractions whose molecular weights lower than 3 kDa, with low concentration (0.025 mg/mL), medium concentration (0.05 mg/mL), and high concentration (0.1 mg/mL), respectively, using Vc as positive control with concentration of 8 μmol/L. The SOD activity in untreated HepG2 cells was approximately 86.63 U/mg protein (U/mgpro). Following H_2_O_2_ induction, intracellular SOD activity decreased significantly to 40.81 U/mgpro, representing only 47.1 % of the level in normal cells.

Treatment with HLPH‐Ⅰ, HLPUH‐Ⅰ, and vitamin C (Vc) solutions markedly restored SOD activity. The highest activities observed were 63.78 U/mgpro (with 0.1 mg/mL HLPH‐Ⅰ), 69.50 U/mgpro (with 0.1 mg/mL HLPUH‐Ⅰ), and 76.14 U/mgpro (with 8 μmol/L Vc), corresponding to increases of 56.28 %, 70.29 %, and 86.59 % (*P* < 0.05), respectively, compared to the model group (MG). Notably, HLPUH‐Ⅰ exhibited a 14 % stronger protective effect on SOD activity than HLPH‐Ⅰ, indicating that in situ dual-frequency ultrasound-assisted enzymolysis enhances the antioxidant efficacy of the hydrolysate and strengthens the cellular antioxidant enzyme system’s capacity to counteract free radical-induced damage. In study of antioxidant activity of β-lactoglobulin by ultrasound and enzymatic treatment, Ma et al. [67] reported that SOD activity increased from 44.95 to 64.26 U/mgprot (ultrasound-treaed 50 °C, pH 8). These findings demonstrate that ultrasound-assisted processing—whether applied to hydrolyzed plant/insect proteins or dairy-derived β-lactoglobulin—significantly enhances the bioactivity of the resulting peptides by improving their capacity to restore intracellular antioxidant enzyme activities, with efficacy further amplified by optimized ultrasonic and enzymatic treatment conditions.

Catalase (CAT) is a heme-containing enzyme, also referred to as a peroxidase, that is present in a wide range of organisms. Its primary function is to catalyze the decomposition of hydrogen peroxide into water and oxygen [68]. As shown in Fig. 7B, intracellular CAT activity in the model group decreased to 4.745 U/mgpro following H_2_O_2_ induction, demonstrating that oxidative stress impaired antioxidant enzyme function and induced cellular damage.

After intervention with HLPH‐Ⅰ, HLPUH‐Ⅰ, and vitamin C (Vc), CAT activity was significantly restored. Under optimal protective concentrations, the highest intracellular CAT activities reached 10.618 U/mgpro (with HLPH‐Ⅰ), 11.616 U/mgpro (with HLPUH‐Ⅰ), and 13.409 U/mgpro (with Vc), representing increases of 123.77 %, 144.81 %, and 182.59 % (*P* < 0.05), respectively, compared to the model group.

Although the antioxidant effects of the HLP hydrolysates were slightly lower than those of Vc, treatment with these peptides restored intracellular CAT activity to 70–85 % of the level in the blank control group. This recovery significantly mitigated H_2_O_2_-induced oxidative damage and confirms the strong antioxidant potential of the enzymolysis products.

Moreover, intracellular CAT activity was higher in cells treated with ultrasound-assisted enzymolysis products (HLPUH‐Ⅰ) than in those non-sonicated products (HLPH‐Ⅰ), with a maximum increase of 13.31 %. This demonstrates that ultrasound-assisted hydrolysates enhance the cellular antioxidant enzyme system by elevating CAT activity, thereby improving the capacity to counteract free radical-induced damage. These results align with the findings of Hu et al. [69] in their study on grass carp scale gelatin antioxidant peptides. They observed that pretreatment with these peptides for 24 h significantly increased the survival rate of HepG2 cells under H_2_O_2_-induced oxidative stress, reduced the apoptosis rate, and enhanced the activities of antioxidant enzymes including SOD and CAT. These findings, along with the results from Hu et al. [69], consistently demonstrate that ultrasound-assisted enzymolysis not only enhances the specific activity of key antioxidant enzymes like CAT and SOD but also provides comprehensive cytoprotective effects under oxidative stress, including improved cell viability and reduced apoptosis, thereby offering a robust strategy for augmenting the efficacy of bioactive peptides in cellular defense mechanisms.

Glutathione peroxidase (GSH-Px) is a crucial intracellular antioxidant enzyme that facilitates the inactivation of hydrogen peroxide and lipid peroxides, thereby preventing their accumulation and reducing oxidative damage to cells [70]. As shown in Fig. 7C, intracellular GSH-Px activity in the model group significantly decreased to 69.87 U/mgpro after H_2_O_2_ induction. Following intervention with high concentrations of HLPH‐Ⅰ, HLPUH‐Ⅰ, and vitamin C (Vc), GSH-Px activity was markedly enhanced, reaching 97.83 U/mgpro, 104.34 U/mgpro, and 119.26 U/mgpro, respectively. These values correspond to increases of 40.02 %, 49.33 %, and 70.69 % (*P* < 0.05) compared to the model group.

Notably, cells treated with ultrasound-assisted enzymolysis products (HLPUH‐Ⅰ) exhibited higher GSH-Px activity than those receiving non-sonicated products (HLPH‐Ⅰ), further demonstrating the efficacy of ultrasonic treatment in enhancing the antioxidant enzyme response. These findings are consistent with those reported by Preety et al. [71], who observed that antioxidant peptide intervention alleviated oxidative damage in HepG2 cells by boosting intracellular antioxidant enzyme activities. The present study, along with the research by Preety et al. (2022), collectively underscores that interventions enhancing intracellular antioxidant enzymes—whether through ultrasound-derived bioactive peptides or herbal extracts like *Tinospora cordifolia*—effectively mitigate oxidative damage and improve cellular resilience. Both approaches demonstrate significant restoration of key antioxidants (e.g., GSH-Px, SOD, CAT) and attenuation of oxidative stress markers, highlighting a shared mechanism of action centered on reinforcing the endogenous antioxidant defense system.

(3) Effects of HLPH-I and HLPUH-I on ROS content in damaged HepG2 cells induced by H_2_O_2_

Under oxidative stress conditions, excessive reactive oxygen species (ROS) can adversely affect cells, potentially leading to cellular damage or death. Studies have shown that certain antioxidants can protect HepG2 cells from oxidative damage by scavenging intracellular ROS [72]. In this study, the intracellular oxidative stress level was assessed using the fluorescent probe 2′,7′-dichlorodihydrofluorescein diacetate (DCFH-DA) [73].

**Fig. 8:**
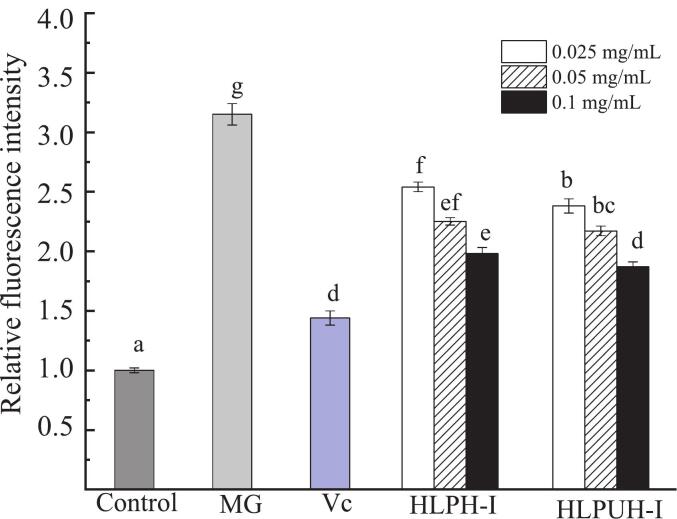
Effects of HLPH-I and HLPUH-I on ROS content in damaged HepG2 cells induced by H_2_O_2_. (Note: MG means model group; HLPH-I, HLPUH-I are HLPH’s and HLPUH’s fractions whose molecular weights lower than 3 kDa, with low concentration (0.025 mg/mL), medium concentration (0.05 mg/mL), and high concentration (0.1 mg/mL), respectively. Using Vc as positive control with concentration of 8 μmol/L. Different letters indicate significant differences, *P* < 0.05).

As shown in Fig. 8, HepG2 cells in the control group exhibited low fluorescence intensity, indicating a basal level of intracellular ROS. Following H_2_O_2_ induction, the fluorescence intensity increased significantly, reflecting elevated ROS levels and successful establishment of an oxidative stress model. In the experimental groups, fluorescence intensity varied markedly among samples and decreased in a concentration-dependent manner. Treatment with 0.1 mg/mL HLPH-I, 0.1 mg/mL HLPUH-I, and 8 μmol/L Vc reduced intracellular ROS levels by 25.43 %, 30.93 %, and 37.83 % (*P* < 0.05), respectively, compared to the model group. These results demonstrate that both HLPH-I and HLPUH-I possess significant intracellular ROS-scavenging capabilities, with the ultrasound-assisted product (HLPUH-I) showing a stronger protective effect.

**Fig. 9:**
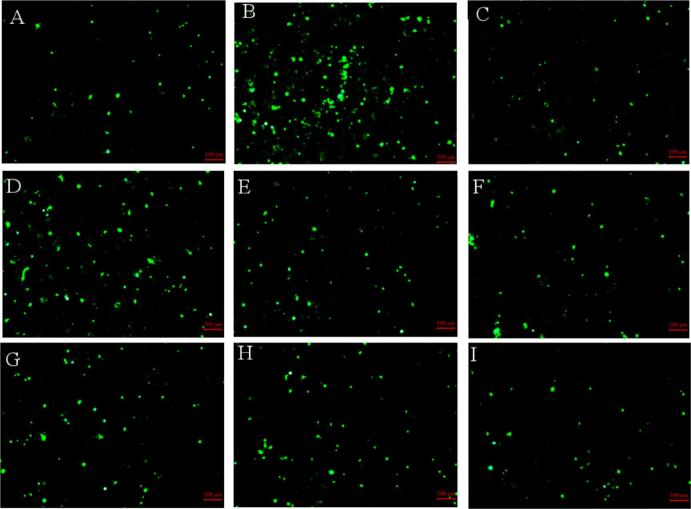
Fluorescence micrographs of HepG2 cells induced by H_2_O_2_ in different samples. (Note: A: Control group, B: Model group, C: Vc (Positive control with concentration of 8 μmol/L), D: HLPH-Ⅰ of low concentration (0.025 mg/mL), E: HLPH-Ⅰ of medium concentration (0.05 mg/mL), F: HLPH-Ⅰ of high concentration (0.1 mg/mL), G: HLPUH-Ⅰ of low concentration (0.025 mg/mL), H: HLPUH-Ⅰ of medium concentration (0.05 mg/mL), I: HLPUH-Ⅰ of high concentration (0.1 mg/mL)).

Meanwhile, fluorescence micrographs in Fig. 9 further support these observations: normal cells exhibited a weak fluorescence signal (Fig. 9A), whereas H_2_O_2_ induction markedly increased fluorescence intensity (Fig. 9B). In the experimental groups, fluorescence intensity decreased in a concentration-dependent manner across both HLPH-I (Fig. 9, Fig. 9) and HLPUH-I (Fig. 9, Fig. 9) treatments.

These results are consistent with the findings of Hu et al. [74], who reported that antioxidant peptides derived from Ankang fish hydrolysate significantly suppressed intracellular ROS in HepG2 cells and effectively alleviated H_2_O_2_-induced oxidative damage. The present study further confirms that HLP enzymolysis products possess notable ROS-scavenging capacity and exhibit strong antioxidant activity. Ultrasound treatment may promote the generation of shorter peptide sequences capable of chelating metal ions, thereby reducing ROS levels. Additionally, Liang et al. [75] observed that ultrasonication altered the spatial conformation of pine nut peptides, enhancing their ability to mitigate H_2_O_2_-induced oxidative damage by inhibiting ROS accumulation and boosting antioxidant enzyme activities in HepG2 cells.

(4) Effects of HLPH-I and HLPUH-I on cell cycle of HepG2 cells induced by H_2_O_2_

The cell growth cycle typically consists of four phases: G_1_ (gap 1), S (synthesis), G_2_ (gap 2), and M (mitosis). Cells possess an intrinsic regulatory mechanism—comprising checkpoint controls at G_0_/G_1_, S, and G_2_/M phases—to ensure each stage is faithfully completed before progression to the next [76]. These checkpoints monitor DNA integrity: minor damage can be repaired, allowing the cycle to continue, whereas severe damage may lead to cell death, preventing further division [77]. Thus, precise regulation of the cell cycle is critical for cell survival.

In this study, flow cytometry was employed to analyze the cell cycle distribution of HepG_2_ cells under oxidative stress conditions, aiming to elucidate the mechanism by which housefly larvae protein enzymolysis products promote cell growth and mitigate damage-induced arrest.

**Fig. 10:**
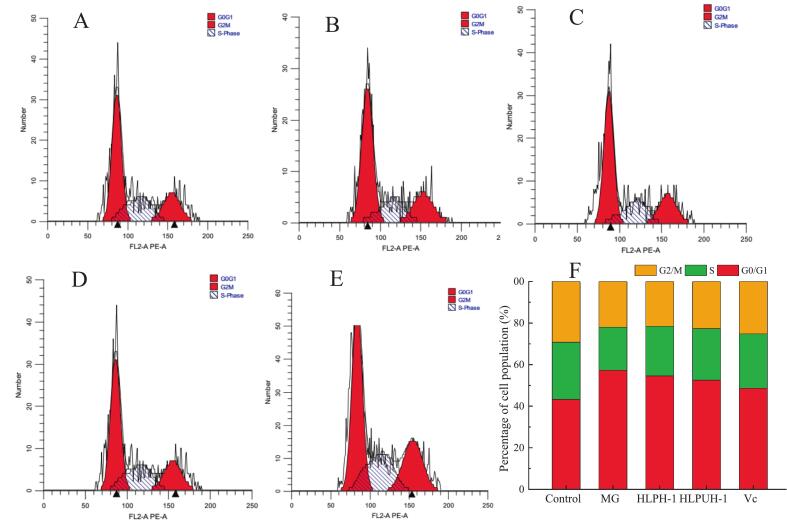
The cell cycles of HepG2 cells analyzed by Flow cytometry. (Note: A: Control group, B: Model group, C: HLPH-Ⅰ of high concentration (0.1 mg/mL), D: HLPUH-Ⅰ of high concentration (0.1 mg/mL), E: Vc (Positive control with concentration of 8 μmol/L), F: percentages of cell population in G0/G1, S and G2/M stages).

As shown in Fig. 10, compared to the control group, the proportion of cells in the G_0_/G_1_ phase in the model (injury) group increased significantly from 43.23 % to 57.29 % (*P* < 0.05), while the population in S phase decreased markedly from 27.56 % to 20.73 % (*P* < 0.05). These results indicate that H_2_O_2_-induced DNA damage triggered arrest in the G_0_/G_1_ phase, preventing cells from progressing to S phase for DNA replication and thereby inhibiting proliferation [78].

Following pretreatment with HLPH‐Ⅰ, HLPUH‐Ⅰ, or Vc, the proportion of G_0_/G_1_ phase cells decreased, and S phase populations increased relative to the model group. This suggests that HLP enzymolysis products can effectively protect HepG2 cells from oxidative DNA damage and support normal cell cycle progression. Notably, HLPUH‐Ⅰ exhibited a stronger protective effect than HLPH‐Ⅰ, which may be attributed to the fact that in situ dual-frequency ultrasound treatment reduces the molecular weight of peptides in the hydrolysate, increases the content of acidic and hydrophobic amino acids, and enhances antioxidant activity. Bi et al. [79] reported a novel peptide, 9R-P201, strongly inhibited the viability, proliferation and migration of liver cancer HepG2 cells and induced apoptosis by down-regulation of FoxM1 expression. These foundings demonstrates that both targeted peptide therapeutics and bioactive hydrolysates can effectively inhibit cancer progression through distinct yet complementary mechanisms. The novel peptide 9R-P201 specifically targets FoxM1 to suppress HepG2 proliferation, migration, and tumor growth, while ultrasound-processed HLPUH‐Ⅰ mitigates oxidative DNA damage and promotes cell cycle recovery. Importantly, HLPUH‐Ⅰ shows enhanced efficacy over conventional hydrolysates, likely due to ultrasound-mediated peptide modification that improves bioactivity.

The promising results of this study highlight several avenues for further research and potential applications. The efficacy and safety of HLPUH‐Ⅰ should be validated using animal models of oxidative stress‐related diseases (e.g., liver injury, diabetes, or aging) to confirm its physiological relevance and therapeutic potential. Additionally, exploring the synergistic effects of HLPUH‐Ⅰ with other natural antioxidants or clinical drugs could lead to combination therapies with enhanced protective outcomes. Further mechanistic studies are also needed to identify specific bioactive peptides within HLPUH‐Ⅰ and elucidate their structure‐activity relationships, cellular targets, and signaling pathways to deepen our understanding of their antioxidant and cytoprotective actions.

## 4 Conclusions

In this study, the effects of in situ 20 ± 2/28 ± 2 kHz dual-frequency ultrasound on the kinetics and thermodynamics of housefly larvae protein (HLP) enzymolysis were investigated. The in vitro antioxidant properties of the resulting hydrolysates were evaluated, and an H_2_O_2_-induced oxidative damage model in HepG2 cells was employed to assess their antioxidant effects.

The results demonstrated that in situ dual-frequency ultrasound (DU) treatment significantly enhanced the enzymolysis process compared to conventional methods: it increased the initial reaction rate, reduced the Michaelis constant (*K_m_*), and lowered the activation energy (*E_a_*), enthalpy change (*ΔH*), and entropy change (*ΔS*). These findings indicate that ultrasound improves the affinity and interaction between the substrate and protease.

The HLP hydrolysates exhibited strong in vitro antioxidant activity and conferred effective protection to HepG2 cells against oxidative stress. Specifically, hydrolysates from ultrasound-assisted enzymolysis enhanced the activities of key intracellular antioxidant enzymes—CAT, SOD, and GSH-Px—and suppressed ROS expression. This study provides a theoretical foundation and technical support for the development of antioxidant peptides derived from housefly larvae protein.

## CRediT authorship contribution statement

**Han Chen:** Writing – original draft, Methodology, Investigation. **Fan Yang:** Writing – original draft, Methodology, Investigation. **Zhuofan He:** Methodology, Investigation. **Liurong Huang:** Methodology, Conceptualization. **Yiming Zhao:** Methodology. **Chunhua Dai:** Methodology. **Ronghai He:** Writing – review & editing, Supervision, Methodology, Investigation, Funding acquisition, Conceptualization. **Haile Ma:** Conceptualization.

## Declaration of competing interest

The authors declare that they have no known competing financial interests or personal relationships that could have appeared to influence the work reported in this paper.
